# Breeding Experience, Alternative Reproductive Strategies and Reproductive Success in a Captive Colony of Zebra Finches (*Taeniopygia guttata*)

**DOI:** 10.1371/journal.pone.0089808

**Published:** 2014-02-27

**Authors:** Nicole M. Baran, Elizabeth Adkins-Regan

**Affiliations:** 1 Department of Psychology, Cornell University, Ithaca, New York, United States of America; 2 Department of Neurobiology & Behavior, Cornell University, Ithaca, New York, United States of America; Columbia University, United States of America

## Abstract

Birds exhibit a remarkable diversity of different reproductive strategies both between and within species. Species such as the zebra finch (*Taeniopygia guttata*) may evolve the flexible use of alternative reproductive strategies, as well as benefit from prior breeding experience, which allows them to adaptively respond to unpredictable environments. In birds, the flexible use of alternative reproductive strategies, such as extra-pair mating, has been reported to be associated with fast reproduction, high mortality and environmental variability. However, little is known about the role of previous breeding experience in the adaptive use of alternative reproductive strategies. Here we performed an in-depth study of reproductive outcomes in a population of domesticated zebra finches, testing the impact of prior breeding experience on the use of alternative reproductive strategies and reproductive success. We provide evidence that older females with prior breeding experience are quicker to initiate a clutch with a new partner and have increased success in chick rearing, even in a captive colony of zebra finches with minimal foraging demands. We also find evidence that the breeding experience of other females in the same social group influences reproductive investment by female zebra finches. Furthermore, we demonstrate that the use of alternative reproductive strategies in female zebra finches is associated with previous failed breeding attempts with the same pair partner. The results provide evidence that age and breeding experience play important roles in the flexible use of both facultative and adaptive reproductive strategies in female zebra finches.

## Introduction

The remarkable diversity of avian reproductive strategies is thought to be determined by variability in ecological resources, opportunities for exploiting social or sexual partners and by phylogeny [1]. A majority of birds form socially monogamous pair bonds and exhibit biparental care. However, molecular evidence suggests that over 85% of socially monogamous species in fact demonstrate the use of alternative reproductive strategies, including extra-pair mating and conspecific brood parasitism, as part of their behavioral repertoire [2]–[4].

Much research has focused on the mechanisms underlying variation in reproductive strategies across species. However, the extent to which individuals exhibit the ability to flexibly adjust their reproductive strategy in response to ecological and social circumstances is still poorly understood. In particular, what sorts of selection pressures select for plasticity in reproductive strategy? Furthermore, what is the role of experience in allowing breeding females to choose an appropriate reproductive strategy for their ecological and social environment?

Strong selection against high failure rates during the first breeding attempt is thought to select for low variability in reproductive outcomes and, thus, a minimal role for experience. However, it is also possible that organisms in unpredictable environments should in fact be flexible and plastic in their reproductive strategies. Indeed, according to a comparative meta-analysis of many avian species, the incidence of two alternative reproductive strategies, extra-pair paternity and egg dumping, were both strongly linked to fast track reproduction [1], [3]. This linkage was found to be especially strong in species with adult mortality rates in excess of 30% and duration of chick feeding of less than 30 days. In addition, there is a positive association between local environmental variability and the use of two alternative reproductive strategies, extra-pair mating and divorce, in socially monogamous passerines [5]. This suggests that there may, in fact, be greater within-species variability in reproductive strategies in species adapted to unpredictable environments.

Like many passerines, zebra finches (*Taeniopygia guttata*) form long-term socially-monogamous pair bonds and display biparental care. They are always paired, even when not actively breeding [6]–[8]. They breed opportunistically in response to unpredictable rainfall and offspring can breed at a very early age (60 days) [7], [9]. In the wild, zebra finches experience very high rates of mortality: in one population the mean annual survivorship for the first 12 months of life was only 4% [7]. The average lifespan of wild zebra finches in two populations was found to range from 53 to 128 days [10], though they can live significantly longer in captivity. Given that the median age of reproduction is 90 days, the vast majority of zebra finches may only get a single opportunity to breed, if any. However, there are many caveats to this data due to the difficulty in tracking the highly mobile populations. It is plausible that, if an individual survives to adulthood, it may live for several years and have many breeding opportunities. Nevertheless, zebra finches have a high rate of clutch failure, both in natural and captive populations [7], [11], [12].

Zebra finches have been found to use alternative reproductive strategies in both field and captive populations, with drastic variation in the rates between different environments. The rate of extra-pair paternity (EPP) is very low in two different field populations (2.4% and 1.7%), but it has been found to be far higher in lab populations (28%, 29%, 15.3%) [13]–[17]. Conspecific brood parasitism (CBP) shows the opposite pattern, with the rate of CBP in the field (10.9% and 5.4%) being somewhat higher than what has been observed in the lab (3.6% and 5.4%) [13]–[15], [18]. Although there is some evidence that individual females may vary in their propensity to engage in extra-pair mating [19], this variability across the lab and field suggests that it is also possible to have plasticity across contexts in the expression of alternative reproductive strategies. Indeed, wild-caught birds breeding in captivity have been found to have a higher rate of extra-pair fertilizations than birds from the same population breeding in the wild (12% versus 1.7%) [17], [14], suggesting that the frequency of alternative reproductive strategies is sensitive to the breeding environment.

Despite their high rates of mortality and rapid development, zebra finches do not fit the classic model of a fast life history species. As opportunistic breeders, they exhibit many adaptations to allow them to survive in a highly unpredictable environment, including timing the onset of breeding to variable environmental cues. They also exhibit permanent rather than short term pair bonds, despite being relatively short lived [20]. Additionally, they show extensive learning in their general social development, including complex socially-guided vocal learning and sexual imprinting [21]–[27].

Thus, what would be the predicted role for experience and reproductive strategies in a bird with this life history? It has been hypothesized that longer developmental periods correspond to increased learning capacities and the development of complex skills in birds, as well as humans [28]–[31]. From this perspective, in rapidly-developing species such as the zebra finch, experience should play a minimal role in reproductive outcomes. However, based on the importance of learning in the general ecology of the zebra finch, we hypothesize that experience plays an important role in breeding, as well.

We performed an in-depth study of reproductive outcomes in a captive population of zebra finches, testing the impact of age and prior breeding experience of females on the use of alternative reproductive strategies and reproductive success. We focused on the role of females, since females are thought to have a stronger influence on reproductive outcomes [32]. We recorded data on pairing, egg laying, chick rearing, and fledging success for 112 inexperienced male and 112 female zebra finches. Sixty females had prior breeding experience and 52 females were inexperienced. To test the impact of the social environment on reproductive outcomes, females were randomly assigned to aviaries in which 1) all of the females were experienced, 2) all were inexperienced or 3) half of the females were experienced and half were inexperienced. To measure the use of alternative reproductive strategies, parents and offspring, including eggs that failed to hatch, were genotyped to determine genetic parentage, which was compared to observational data to identify incidences of extra-pair fertilization (EPF) and conspecific brood parasitism. We predicted that females with prior breeding experience would be more skilled parents and more likely to strategically use extra-pair mating to improve their reproductive success.

The role of experience in shaping reproductive strategies and breeding outcomes is still poorly understood. Across many species of birds, there is a strong statistical association between age and breeding success [33]–[36]. One plausible explanation is that older birds may have developed certain skills important either in survival and self-maintenance more generally (such as foraging ability) or in skills specifically related to breeding (such as incubation, chick rearing, or nest defense) [34], [35]. However, in natural populations, age is almost always correlated with experience, making it difficult to disentangle general age effects from learning or from some physiological change from past breeding.

Although there is very little overlap in age between experienced and inexperienced females in our sample, the present study represents an improvement over many previous investigations of the effect of age on breeding success for several reasons. First, we are able to control environmental variability by using a captive population in naturalistic social aviaries. Second, we are able to control, to a limited extent, the contributions of the pair partner, since both temporal constraints related to pair formation and breeding experience of the partner may impact the reproductive success of the pair. Finally, we are able to test the impact of variation in the social environment by experimentally manipulating the breeding experience of the other birds in the same social group.

## Materials and Methods

### Subjects

The male and female birds used as parents in this study were drawn from several different populations within two domesticated aviary colonies at Cornell University. Birds from these populations varied on several dimensions, including age (and whether information was available about their age) and whether or not information was known about their parentage. To control the composition of birds within breeding aviaries, birds from different populations were divided into blocks and then randomly assigned to aviaries. If information was known about the parentage of birds, possible siblings were randomly assigned to different treatment aviaries. The diet throughout was *ad libitum* Kaytee Forti-Diet Pro-Health Finch feed, grit, cuttle bone, and water.

Females ranged in age from approximately 62 days to 421 days, with an average age of 205±132 days. All males in the study were inexperienced breeders and had been housed in single-sex aviaries since 40-50 days old. Males ranged in age from 60 days to 562 days old at the start of the study, with the average age of 219±180 days.

Exact hatch dates were known for 71/112 (63.4%) of the females and 97/112 (86.6%) of the males; recording hatch dates was not standard practice in the facility prior to the start of the study. However, fledglings are removed from their natal aviary at around 40–50 days of age, when sexually dimorphic plumage first becomes apparent. At this time, they received a numerical metal band (except one male with a known hatch date). Bands are assigned to individuals in numerical order, meaning that there is a strong correlation between ID number and age (*F*_1,166_ = 53.31, *p*<0.0001, *r* = 0.49). Thus, we used numerical ID to predict age, when the exact age was not known.

All animal procedures conformed to Federal and State regulations and were approved by the Cornell University Institutional Animal Care and Use Committee (Protocols 2007-0074 and 2008-0001).

### Experimental Setup

Within two weeks prior to the start of the study, the mass, tarsus lengths (in triplicate), and a tissue sample (either blood or pin feather) was collected from all parents in the study. Masses were regressed on tarsus length within each sex to produce a mass-tarsus residual score, a measure of body condition. Previously-paired females were visually- and acoustically-isolated from all males for one month prior to the start of the study, presumably including all former partners, to facilitate rapid pairing with a new male.

Females were then randomly assigned to an aviary in one of three treatments: an aviary in which all females had prior breeding experience (all-experienced, *n* = 5 aviaries), all females were inexperienced (all-inexperienced, *n* = 4 aviaries), or an aviary in which there was a 50∶50 mix of experienced and inexperienced breeders (mixed, *n* = 5 aviaries). This created four aviary treatment types: experienced females in all-experienced aviaries, inexperienced females in all-inexperienced aviaries, experienced females in mixed aviaries (mixed-experienced), and inexperienced females in mixed aviaries (mixed-inexperienced). Thus, there were a total of 60 experienced females, 52 inexperienced females, and 112 inexperienced males used as parents in the study. Experienced females had previously been housed in a breeding aviary with males and had the opportunity to pair and breed, but details of their breeding outcomes were not known.

At the start of the study, sixteen adults (8 females, 8 unfamiliar males) were released into one of 14 breeding aviaries, each equipped with eight nest boxes and coconut husk (for nest building). The birds were given the opportunity to pair and breed for a total of 60 days. The first 35 days of the study is referred to as Phase One. Thirty-five days is the minimum length of time in which a pair can pair start a clutch, fledge chicks, and initiate a second clutch, and thus the period in which breeding experience treatment could be guaranteed for the full aviary. All eggs laid between 35 and 60 days were considered to be a part of Phase Two. In Phase Two, all eggs were removed from the nests on the day of hatching and artificially incubated. Eggs that had been laid during the first 35 days but had not yet hatched were allowed to remain in the nest and develop normally. In Phase Two, tissue samples were collected from eggs after 5–8 days of artificial incubation for genotyping. Phase Two provided an opportunity to measure the use of alternative reproductive strategies as a function of reproductive outcomes in Phase One, without the substantial loss of samples due to egg mortality.

### Nest Checks

Throughout the study (May 25, 2010 to July 31st, 2010), nests were checked daily between 9am and 11am. The presence of eggs, hatchlings and fledglings was recorded. We also recorded egg condition: buried or not; cracked, missing, or discolored. Eggs were marked on the day laid with pencil. Chicks were marked on the limbs and posterior down feathers with colored permanent marking pens and color-banded at approximately 12 days of age. Previous research in our lab suggests that this does not affect hatchling mortality or parental acceptance. The mass of all chicks was recorded each day until fledging. Any eggs or chicks found dead outside of the nest were collected and, if the identity of the egg could be determined, were also genotyped. The final status of all eggs laid during the study was recorded (broken, buried, egg found outside nest or missing altogether, failed to hatch, nestling death, fledged, and broken in handling).

### Observations

There were two types of observations performed. Each morning between the hours of 7am (lights on) and 9am, random focal observations were performed for periods of 5 minutes. This is a period of high activity in the zebra finches and it also the time during which females are predicted to be laying [37]. There were 1050 individual observation periods, amounting to 89 hours of observation. Each bird was observed an average 4.76±1.37 times. The bird's location in the aviary as well as the infrequent incidents of aggressive behaviors (attack, chase, beak fence, jabbing, supplant, threat call) were recorded in JWatcher v. 1.0 by observers sitting in front of the aviary. Between the hours of 12-4pm, observations were performed to determine pairing status and nest box occupation, based on clumping, allopreening and two birds occupying the nest box together. These observations were performed throughout pairing, egg laying and chick rearing, until pairing status was confirmed by multiple independent observers across several observational periods. After pairing status was determined, the proportion of time spent in the nest box, on the perch of the nest box, and on the floor of the aviary was tabulated for the period after the first egg was laid.

### Genotyping procedure

To determine genetic parentage, all parents and offspring were genotyped at six highly-polymorphic microsatellite loci selected from Forstmeier et al., 2008 [38], based on non-overlapping size ranges, the absence of null alleles, and similar PCR programs. Three primers were labeled in using 6FAM fluorescent tags (*Tgu12*, *Tgu9*, and *Tgu1*) and three were labeled using NED fluorescent tags (*Tgu4*, *Tgu3*, and *Tgu8*).

Genomic DNA was extracted and purified using either the Qiagen DNEasy Blood and Tissue kit (blood and pin feather samples) or Agencourt DNAdvance kit (pin feather and nestling egg/tissue samples). PCR amplifications were performed using the QIAGEN Type-It Microsatellite Kit (QIAGEN, Cat. No. 206243) to perform a multiplex PCR reaction containing all six primer pairs. Each 25-µL PCR contained 12.5-µL of the 2x Type-it Multiplex PCR Master Mix, 2.5-µL 10x primer mix (containing 2 µM each primer), 2-µL template DNA, and 8-µL ddH2O. The following is the PCR program used: an initial hot-start 5-min denaturation step at 95°C; followed by 5 cycle touchdown: 94°C for 30 s, 60–56°C for 90 s (dropping 1°C per cycle), 72°C for 30 s; another 23 cycles 94°C for 30 s, 56°C for 90 s, 72°C for 30 s; and a final extension at 60°C for 30 min. PCR products were analyzed on an Applied BioSystems 3730xl Genetic Analyzer. Raw data were analyzed using Genemapper 4.0 software.

Parentage analysis was performed in Cervus, which assigns the most likely candidate parent of each sex from the known parent genotypes within each aviary [39]. A total of 1368 eggs were laid during the two studies, 639 in Phase One and 729 in Phase Two. Genotypes could be established for 938 (68.6%) samples (see Table 1).

**Table 1:** Egg Outcomes and Genotyping Success.

	*Successful Genotyping*		*No Attempt/ Failed*	
Final Status	Phase One	Phase Two	Phase One	Phase Two
**Failed to Hatch**	49	-	52	-
**Nestling Death**	73	-	0	-
**Fledged**	118	-	0	-
**Buried**	16	-	102	-
**Outside Nest**	18	-	95	-
**Broken**	28	-	45	-
**Broken in Handling**	8	-	31	-
**Incubated - Failed to Develop**	-	92	-	83
**Incubated - Embryo**	-	536	-	9
**No sample**	-	-	4	9
***Total Numbers***	*310*	*628*	*329*	*101*
Parentage Assigned w/ 95% confidence	281	568	-	-
Compared to Observed Parentage	272	525	-	-

Eggs listed under ‘Successful Genotyping’ were genotyped at a minimum of 5/6 loci. ‘No Attempt/Failed’ refers to eggs that we were not able to successfully genotype, either due to lack of sample or multiple failed genotyping attempts. In both cases, the number of eggs in each final status category is listed: Failed to Hatch, Nestling Death, Fledged, Buried, Outside Nest, Broken, Broken in Handling and No Sample in Phase One and Incubated – Failed to Develop and Incubated – Embryo and No Sample in Phase Two. For eggs that were successfully genotyped, the number of eggs for which parentage was assigned with 95% confidence and the number of eggs that could be compared to observed parentage are listed.

In Phase One, genotypes could be assigned for 310 out of 628 eggs (48.4%). Genetic parentage could be established with 95% confidence for 281 samples. This could be compared to observational data about the expected parents for 272 samples. Following Schielzeth and Bolund (2010), we defined two main categories of eggs: eggs that were laid in the female's own nest and incubated by her or her social partner (‘own nest’) and eggs that were laid in another nest and not incubated by the female or her partner (‘other nest’) [18]. There were only 15 cases of eggs found in an “other nest.” Four cases appeared to be linked to dispute over nest ownership and in one case the egg was laid in an inactive nest. Thus, there were only 10 clear cases of conspecific brood parasitism (CBP). Five of those cases were found to be quasi-brood parasitism, in which the observed male owner was the genetic parent, but not the observed female. There were 32 cases of extra-pair paternity out of 272 eggs (11.7% rate), not including the cases of quasi-brood parasitism.

In Phase Two, genotypes could be assigned for 628 out of 729 eggs (86.1%). Genetic parentage could be established with 95% confidence for 568 individual eggs laid. Observed parentage could be compared to the predicted parentage for 525 individuals. In total, 100 eggs were found to have been laid in an “other nest”, though 9 of those cases were linked to dispute over nest ownership. This category of “other nest” cannot be subdivided in Phase Two because the eggs were removed on the date they were laid and were not replaced with dummy eggs. Thus, they could not be incubated by the nest owner, so it is not clear whether these ‘other’ eggs represents CBP attempts, ‘egg dumping’, or, more likely, the start of a new nest. In addition, there were 49 cases of EPP out of 525 eggs (9.3%).

### Heterozygosity

We used the microsatellite genotypes to generate a measure of heterozygosity, multilocus heterozygosity (MLH), calculated as the proportion of heterozygous loci within an individual [40]–[43]. An arcsine transformation was used to analyze MLH data.

### Statistical Analyses

All statistical analyses were performed in R v. 2.1.5.1. Non-parametric statistics (Kruskal-Wallis tests or Spearman's ρ) were used to analyze egg and chick count data, which were not normally distributed and could not be modeled using other parametric statistics. Several variables, such as pair formation or whether an egg was a result of a within- or extra-pair fertilization, were coded as nominal data and were analyzed using a generalized linear mixed model with a binomial link function (GLMM; *glmer* in R package *lme4*) with female parent population (cohort) included as a random factor. To analyze the relationship between the probability that a given egg hatched or fledged, we used a GLMM with a binomial link function with female parent identity included as a random factor. Mass-tarsus residuals, age, days to clutch initiation, proportion of time spent in nest box, and arcsine MLH were all introduced as continuous variables. To analyze the relationship between days to first egg and experience, we used a linear mixed model with both aviary and room included as random factors (LMM; *lmer* in R package *lme4*). To test whether EPF eggs differed in heterozygosity, we used a LMM with the genetic female parent identity included as a random factor. When performing LMM or GLMM, we used a likelihood ratio test (LRT) to compare the full model to a reduced null model with only the factor of interest removed to test for significance of the fixed effect.

## Results

### Pair Formation

**Figure 1:**
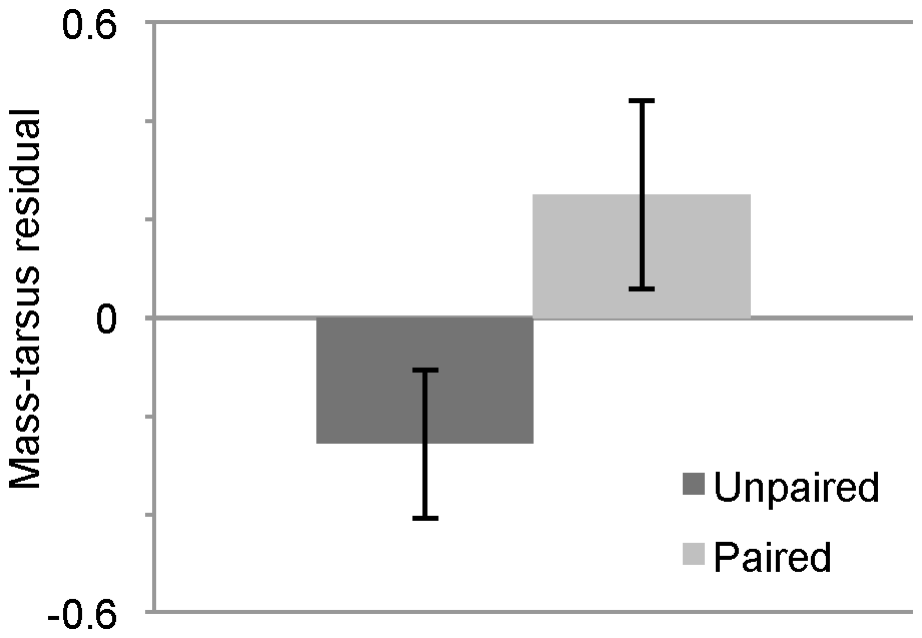
Female pairing status by body condition. Mean female mass-tarsus residual ±SE, a measure of body condition, for 112 females, depending on whether or not they formed a clear pair with a male.

Only 55 out of 112 females (49%) formed clear pairs with males during the first 35 days (two females formed a same-sex pair). Neither age nor experience predicted whether or not females formed a clear pair (age: GLMM with binomial errors, *χ* ^2^(1) = 0.00055, *p* = 0.98; experience: GLMM with binomial errors, *χ* ^2^(1) = 0.031, *p* = 0.86). However, female mass-tarsus residual, a measure of body condition, was a predictor of pair formation (GLMM with binomial errors, *χ* ^2^ = 3.88, *p* = 0.049; Fig. 1). Although there was a strong correlation both between female mass-tarsus residual and age (*F*_1,110_ = 23.31, *p*<0.0001, *r* = 0.42) and female mass-tarsus residual and breeding experience (*F*_1,110_ = 27.31, *p*<0.0001, *r* = 0.45) in our sample, only mass-tarsus residual remained a significant when compared to a reduced model including all other predictors (GLMM with binomial errors with age, breeding experience and mass-tarsus residual as predictors and parent cohort included as a random effect, LRT for mass-tarsus residual: *χ* ^2^(1) = 5.25, *p* = 0.022, LRT for age: *χ* ^2^(1) = 0.0034, *p* = 0.95, LRT for experience: *χ* ^2^(1) = 0.55, *p* = 0.46).

There was a positive correlation between a female's age in days and her partner's age (*F*_1,53_ = 6.76, *p* = 0.012, *r* = 0.33). However, there was no correlation between a female's mass-tarsus residual and the mass-tarsus residual of her male partner (*F*_1,53_ = 0.52, *p* = 0.48).

### Reproductive Success

A total of 638 eggs were laid during the 35 day period of Phase One. A detailed breakdown of egg outcomes and whether or not eggs were successfully genotyped can be found in Table 1. A total of 191 eggs hatched, but 73 of those chicks (38%) died prior to fledging. Thus, only 118 chicks survived until fledging—a fledging success rate of only 18.8%. However, the success rate was higher for the eggs that remained in the nest (118 out of 240 eggs, 49.2%). Of the 729 eggs laid in Phase Two (see Table 1), 545 (74.7%) appeared to develop normally in the artificial incubator. The remainder did not develop normally and were either unfertilized or the embryo died within the first few days of development (no data was collected for 9 eggs). Of the 175 eggs that failed to develop, 92 (52.6%) were successfully genotyped.

Given that a female paired, none of the measures were significant predictors of egg or chick numbers in Phase One. Female breeding experience did not predict the number of hatched or fledged chicks in the nest (hatched: Kruskal-Wallis test, *χ* ^2^ = 0.17, *p* = 0.68; fledged: Kruskal-Wallis, *χ* ^2^ = 0.69, *p* = 0.41). Female age did not predict the number of hatched or fledged chicks (hatched: Spearman ρ = 0.071, *p* = 0.60; fledged: Spearman ρ = 0.14, *p* = 0.31). Female mass-tarsus residual not a significant predictor of the number of hatched chicks in the nest (Spearman ρ = 0.004, *p* = 0.98) or the number of fledged chicks (Spearman ρ = 0.094, *p* = 0.49).

However, there was a significant difference in the hatching success of the eggs of experienced and inexperienced females, controlling for female as a random factor (GLMM with binomial errors, *χ* ^2^(1) = 10.89, *p* = 0.00097). Inexperienced females in fact hatched a higher percentage of eggs than experienced females (38.3% versus 30.5%), though they laid fewer eggs overall (217 versus 338). Additionally, there was a non-significant trend suggesting that an egg hatched in the nest of an experienced female was more likely to fledge, again controlling for female as a random factor (GLMM with binomial errors, *χ* ^2^(1) = 2.95, *p* = 0.086). A larger proportion of the eggs that hatched in the nest of experienced females fledged in comparison to eggs in the nest of inexperienced females (71.7% versus 57.3%).

**Figure 2:**
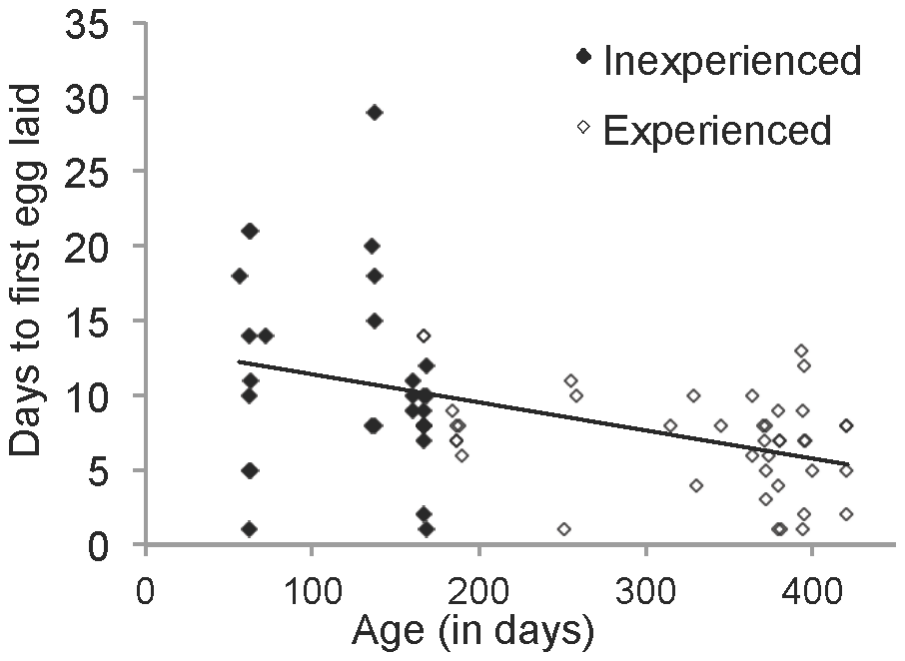
Days to clutch initiation by age and breeding experience. Each data point represents a single female (*N* = 71) that laid an egg during Phase One. Females with no prior breeding experience are shown with the closed symbols and females with breeding experience are shown with open symbols. The line depicts the fitted linear regression; the analysis used to test for significance was a linear mixed model controlling for aviary and room as random factors.

Females with prior breeding experience initiated their clutch (laid the first egg in nest) on average 2.3 days faster than inexperienced females (LMM, *χ* ^2^(1) = 10.82, p = 0.0010) with both aviary and room included as random effects. This relationship remains significant when controlling for mass-tarsus residuals (LMM, *χ* ^2^(1) = 9.65, *p* = 0.0019). There was a strong negative association between age and the days to clutch initiation (LMM, *χ* ^2^(1) = 11.80, p = 0.00059), including aviary and rooms as a random factor (Fig. 2). Furthermore, this association exists even when very young birds (younger than 90 days) are excluded from the analysis (LMM, *χ* ^2^(1) = 9.21, p = 0.0024). Thus, this result is not driven by presence of very young birds in the data set.

**Figure 3:**
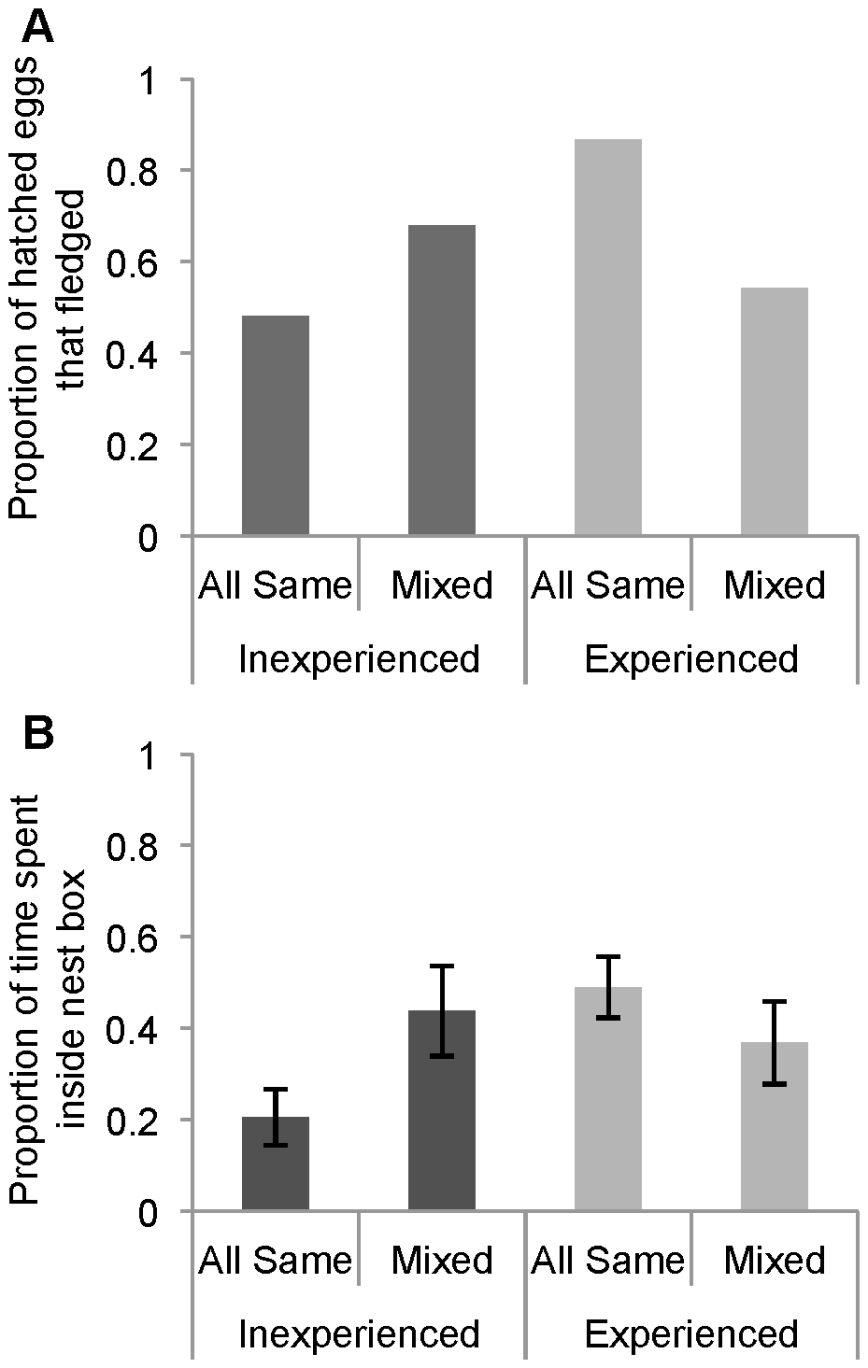
Relationship between aviary treatment type and A) the proportion of the hatched eggs that fledged for females in each of the four aviary treatment types (*N* = 174 hatched eggs with a known female parent) and B) the mean ±SE proportion of time females spent inside the nest box after the first egg was laid (*N* = 49 females assigned to a nest box). There are four aviary treatment types: inexperienced females in all-inexperienced aviaries (All Same-Inexperienced), inexperienced females in mixed aviaries (Mixed-Inexperienced), experienced females in all-experienced aviaries (All Same-Experienced), and experienced females in mixed aviaries (Mixed-Experienced). Results from inexperienced females are shown in dark grey and experienced females in light grey.

The aviary treatment type (all-experienced, all-inexperienced, mixed-inexperienced, mixed-experienced) did not impact the number of hatched or fledged chicks, given that a female had paired (Kruskal-Wallis, hatched: *χ* ^2^(3) = 1.40, *p* = 0.70; fledged: *χ* ^2^(3) = 1.38, *p* = 0.71). However, there was a significant interaction between aviary treatment type and experience in the fledging success of hatched eggs, controlling for both aviary and room as random factors (GLMM with binomial errors, χ^2^(1) = 5.24, *p* = 0.022) (Fig. 3A). Eggs hatched in the nests of experienced females in all-experienced aviaries were more likely to fledge than eggs hatched in the nests of experienced females in mixed aviaries. In contrast, eggs hatched in the nests of inexperienced females in mixed aviaries were more likely to fledge than eggs hatched in the nests of inexperienced females in aviaries in which all the females were inexperienced.

### Observational Data

In females who paired, aviary treatment type was a significant predictor of the proportion of time a female spent inside the nest box controlling for treatment cage and room as random effects, presumably incubating their eggs or brooding chicks (LMM, χ^2^(1) = 4.55, *p* = 0.033, Fig. 3B). Inexperienced females in all-inexperienced aviaries spent less time in their nest box than all other treatment groups, whereas experienced females in all experienced aviaries spent significantly more time in their nest box. No other behavioral measure was significantly different among aviary treatment types or associated with any other measures.

### Alternative Reproductive Strategies

We found a very small number of cases of conspecific brood parasitism in Phase One (10 out of 272 eggs, 3.7%) and all of these were from only two females. Because the number of CBP cases was so small, it is not possible to test hypotheses about what influenced the rate of CBP. However, there was a much higher rate of extra-pair fertilizations (EPF): 32 out of 272 eggs (11.7%) in Phase One and 49 out of 525 eggs (9.3%) in Phase Two were from EPFs. This was a fairly common reproductive strategy, as 15 out of the 73 females who laid eggs (20%) laid at least one egg fertilized by an extra-pair male in Phase One.

### Predictors of EPFs

Female breeding experience, female age, female body condition and male body condition were not significant predictors of whether a female laid an EPF egg in Phase One. Thus, we tested several hypotheses related to offspring quality which may indicate that females obtain genetic benefits from extra-pair eggs. The following analyses were performed using generalized linear mixed effects models, including whether egg was an EPF egg as a fixed effect and female parent identity as a random effect. Extra-pair chicks did not appear to be better on the measures of quality that we collected. Extra-pair eggs were no more likely than within-pair eggs to hatch (GLMM with binomial errors, *χ* ^2^(1) = 1.28, *p* = 0.26) or fledge (GLMM with binomial errors, *χ* ^2^(1) = 0.51, *p* = 0.47). EPF chicks did not weigh more at fledging (LMM, *χ* ^2^(1) = 1.57, *p* = 0.21). Extra-pair eggs were also no more likely to develop normally when artificially incubated (GLMM with binomial errors, χ^2^(1) = 1.02, *p* = 0.31), a stronger test of the indirect benefits hypothesis of extra-pair mating.

However, extra-pair offspring may benefit from increased genetic variability, consistent with the heterozygosity theory of mate choice [43]. There was a non-significant trend suggesting that extra-pair eggs were more heterozygous than within-pair eggs using the arcsine-transformed multilocus heterozygosity (MLH) and controlling for female parent identity as a random effect (LMM, χ^2^(1) = 3.42, *p* = 0.064) [40]. However, heterozygosity was not a significant predictor of hatching (LMM, χ^2^(1) = 0.91, *p* = 0.35) or fledging success (LMM. χ^2^(1) = 0.12, *p* = 0.73).

In Phase Two, the EPF rate among females who had a failed breeding attempt in Phase One was 39 out of 243 eggs (16%) versus 10 out of 144 eggs (6.9%) among females that had been successful. There was a nearly significant negative association between the probability that a Phase Two egg was fertilized by an extra-pair male and the number of fledged chicks in the female's nest in Phase One, controlling for female as a random factor (GLMM with binomial errors, *χ* ^2^(1) = 3.73, *p* = 0.054). There were no clear trends indicating which causes of nest failure (aborted nests, hatching failure or nestling deaths) may have led females to pursue extra-pair matings in our data.

## Discussion

### Reproductive Outcomes

Consistent with previous studies in zebra finches, we find that even in a captive colony with minimal foraging demands, there is a great deal of variability in breeding success [12]. This degree of variability is especially remarkable in a species with high-mortality and fast development, where there is expected to be very strong selection against high failure rate in reproduction.

The observed rate of pair formation was low, though not markedly different from that found in previous studies in our lab using similar numbers of birds in pairing aviaries [20]. In a study in which behavioral evidence of pair formation between two males and two females was measured, the failure to form a clear pair bond was associated with the two males courting one female in preference over the other [44]. In this context, the finding that female mass-tarsus residual is the only predictor of pair formation perhaps suggests that males prefer to court and pair with females in better body condition, leaving females in poor condition without a partner [45].

We also find evidence that, within pairs, zebra finches appear to mate assortatively by age. Assortative mating by age has been found in a number of avian species [46]–[48], though it has not previously been observed in zebra finches, to our knowledge. Often this assortative mating is attributable to structural factors, such as younger individuals arriving at the breeding grounds later in the season or forming pairs with individuals who reach reproductive maturity around the same time. Since the timing of the initiation of pairing was controlled, no such temporal factors can explain the assortative mating observed in our study. Furthermore, the lack of correlation between body condition of male and female partners suggests that zebra finches may be using alternative cues for age rather than condition to pair assortatively.

Despite the variability in reproductive outcomes, there are few reliable predictors of reproductive success. However, we find several ways in which breeding experience and age each appear to provide significant reproductive benefits. First, inexperienced/younger females lay fewer eggs (possibly due to the later initiation of breeding), but have a slightly higher hatching success relative to experienced/older females. However, there is a non-significant trend suggesting that older females with prior breeding experience are more likely to be successful in raising hatched chicks to survival. Additionally, older/experienced females are significantly more successful at raising chicks to fledging when housed in aviaries with other experienced females. These results suggest that the greatest benefit of age and experience may come during the chick rearing phase, rather than during incubation, where particular skills, such as feeding or brooding, improve with experience. However, this trend requires further exploration.

Older/experienced females also initiate clutches sooner. This result suggests that either age or experience prime the female to produce eggs more quickly. It is also possible that older or experienced females progress more quickly through the courtship, pairing and nest-building phases of breeding, despite the inexperience of their partners. In a previous study, individuals breeding a second time with a previous partner also initiated clutches approximately three days faster than birds who were experimentally forced to re-pair [20]. Thus, it remains to be tested whether the observed acceleration in egg laying is a function of behavioral or physiological readiness of the female. Nevertheless, given the importance of rapid reproduction in opportunistic breeders such as the zebra finch, faster clutch initiation is likely to be an ecologically-relevant benefit.

Finally, the impact of the breeding experience of other females within the aviary (aviary treatment type) provides suggestive evidence that social interactions between experienced and inexperienced females may impact reproductive outcomes for both. Given that females were randomly assigned to all-same or mixed aviaries, any differences between groups can be attributable to social factors. Inexperienced females, in particular, seem to benefit from sharing an aviary with experienced females, substantially increasing their fledging success. Inexperienced females in mixed aviaries are indistinguishable from experienced breeders in the amount of time spent inside their nest box. This suggests that they may spend more time incubating when in a social group with more experienced females. Experienced females, however, appear to do worse when in an aviary with inexperienced females. It is unclear what particular changes in the social environment may have led to these different outcomes, but an intriguing possibility that inexperienced breeders may learn how to be better parents by observing conspecifics. Another possibility, based on the differential-allocation hypothesis, is that females vary their parental investment in response to their relative desirability as mates [49], [50]. If they are in a social group in which all females are older, more experienced and in better body condition, they may increase their parental investment in order to more effectively compete for mates, whereas females in social groups with more variability do not need to differentially allocate parental investment. These hypotheses remain to be more fully tested, however.

### Extra-pair paternity

The rate of extra-pair paternity in this study (81 out of 797 total eggs, 10.2%) is consistent with, though on the low end of, other studies in captive populations of zebra finches [13]–[16], [18]. One possible reason for the higher rate in captive populations more generally is that these birds face a substantially different social environment than field populations. These differences may include, but are certainly not limited to, breeding at higher densities than what is found in the field. Additionally, individuals are in constant proximity to the nest, since they cannot leave the nesting site to forage as they would in the wild. Thus, higher rates of extra-pair paternity in the lab are consistent with the observation that EPP occurs at a higher frequency when individuals nest at higher densities [51]–[54].

Nevertheless, the plasticity of alternative reproductive strategies, including the significant differences across field and lab populations, suggests a great deal of flexibility in reproductive behaviors. Future research should investigate the specific cues and environmental factors that may underlie the flexible adjustment of reproductive strategies. This is especially important because the zebra finch has become a model organism, commonly studied for its social and reproductive behavior in the lab [55].

None of the measured female characteristics were associated with an increased rate of extra-pair paternity among females. One reason for this may be that the rate of extra-pair paternity is in fact more related to intrinsic features of the female unrelated to age, breeding experience or body condition [19].

However, we tested several hypotheses related to offspring quality which may indicate that females may obtain genetic benefits from extra-pair eggs. Although extra-pair eggs did not appear to benefit from increased growth or survival before or after hatching, extra-pair offspring may benefit from increased genetic variability, consistent with the heterozygosity theory of mate choice [43]. Microsatellite heterozygosity has been found to be correlated with measures of health and survival in multiple species (see [56] for review), though the correlations are generally quite weak [42]. Nevertheless, the trend suggesting that extra-pair offspring are more heterozygous than within-pair offspring suggests either that females are mating with extra-pair males with higher overall heterozygosity [57], [58] or that females are mating selectively with extra-pair males who are more dissimilar from themselves than their social mate [59]. Another intriguing possibility is that females mate multiply and use a ‘genetically loaded raffle’ to ensure that their extra-pair offspring benefit from increased heterozygosity [60].

Furthermore, unlike a recent experimental study, we find evidence suggesting that female zebra finches who failed to successfully fledge a chick in Phase One laid a higher proportion of EPF eggs in a second breeding attempt, although the trend did not reach significance [61]. There are several possible reasons this outcome may differ from previous findings. Our measure of reproductive failure was the failure to successfully raise at least one chick to fledging during the first breeding attempt, as opposed to hatching success. Failure to fledge any chicks may be a more robust predictor of switches in reproductive strategies than hatching failure. Second, because we did not experimentally manipulate nesting failure, females in this study had the benefit of the full complement of natural cues related to her male partner and his quality that may have directly resulted in the failed breeding attempt. This finding that experience from one breeding attempt may influence behavior during the second attempt leaves open the possibility that learning influences reproductive investment and the flexible use of alternative reproductive strategies in zebra finches.

However, given the observational nature of this study, another plausible explanation is that females who mated multiply during Phase One were also more likely to have lower hatching success, perhaps due to less help from the pair partner. As a result, we may have obtained a biased sample of eggs from promiscuous females during Phase One, leading to the association between fledging failure in Phase One and promiscuity in Phase Two. Because of the sampling method, we do not have the ability to disentangle these two explanations with the current data.

There are several limitations to this study. First, because there is very little overlap in age between experienced and inexperienced females in our sample, we are not able to disentangle the effects of age and experience. A fully experimental design, in which age is not a confound, is still needed to confirm these tentative findings. Second, we performed many tests on this data set, but we chose not to correct for multiple testing. Some findings would not remain significant if we chose a p-value of less than 0.05, but because this was an exploratory study we have presented these findings with an uncorrected p-value. Finally, this work was performed on a population of domesticated zebra finches, whose life history differs from wild populations in significant ways. Clearly, the ecological and social environment in which the birds are breeding can have a significant impact on reproductive outcomes, which means that extrapolation from lab populations to the field is risky. Nevertheless, these findings provide evidence consistent with previous work in zebra finches and suggest several avenues for future research on the role of experience in breeding outcomes.

## Conclusions

Consistent with recent evidence and theories regarding the evolution of flexible mating strategies, we find evidence that age and breeding experience impact reproductive outcomes in the zebra finch. Older and experienced females initiate egg laying faster and appear to be more successful at rearing chicks until fledging, though inexperienced females have slightly better hatching success. The social environment matters as well, with the breeding experience of other birds within the same social group influencing reproductive outcomes. Females also appear to use information about the success of one breeding attempt to make decisions about a second attempt, potentially switching strategies and pursuing adaptive extra-pair mating when the first attempt was unsuccessful. Future research should investigate the specific mechanisms by which experience influences reproductive outcomes, particularly to test whether the improvement in reproductive success is a result of learning, physiological changes or some other mechanism.
